# Cell adhesion molecules’ altered profile in benign and malignant salivary gland tumors. The paradigm of beta4-integrin, desmoglein-2, ICAM-1 and CD44s

**DOI:** 10.1186/s40709-020-00130-5

**Published:** 2020-12-07

**Authors:** Dimitrios Andreadis, Athanasios Poulopoulos, Apostolos Epivatianos, Alexandros Nomikos, Dimitrios Parlitsis, Konstantinos Christidis, Calypso Barbatis, Dimitrios Kavvadas, Alexandros Toskas, Theodora Papamitsou, Dimitrios Antoniades

**Affiliations:** 1 Department of Oral Medicine/Pathology, School of Dentistry, Aristotle University of Thessaloniki, Thessaloniki, 54124 Greece; 2 Department of Oral Medicine/Maxillofacial Pathology, School of Dentistry, Aristotle University of Thessaloniki, Thessaloniki, 54124 Greece; 3 Department of Histopathology, Asklipion” Hospital of Athens, Athens, 10564 Greece; 4 Department of ENT Surgery, Mitera” Hospital of Athens, Athens, 15123 Greece; 5 Pathology, External Consultant, HISTO-BIO-DIAGNOSIS-HBD, Athens, 11526 Greece; 6 Laboratory of Histology and Embryology, School of Medicine, Faculty of Health Sciences, Aristotle University of Thessaloniki, GR-54124 Thessaloniki, Greece

**Keywords:** Cell adhesion molecules, Salivary gland tumors, Immunohistochemistry

## Abstract

**Background:**

Alterations in intercellular and cell-extracellular matrix connections contribute to tumour development. This study investigates the expression of specific cell adhesion molecules (CAMs) in salivary gland tumors (SGTs).

**Methods:**

Formalin–fixed, paraffin– embedded tissue specimens of different types of 34 benign and 31 malignant SGTs and normal salivary glands were studied using Envision/HRP immunohistochemical technique for Desmoglein-2 (Dsg-2), beta4-integrin, CD44s and ICAM-1. Intensity of staining was evaluated in a semi-quantitative manner. Results were analyzed using Kendall’s τ and Spearman’s ρ as correlation criteria.

**Results:**

Dsg-2 in intercellular space, beta4-integrin in cell-basal membrane, and CD44s in both types of contacts were strongly expressed in normal acinar and ductal cells, whereas ICAM-1 was expressed only at the endothelium and sparse stromal cells and monocytes. Strong correlation was found between Dsg-2 expression in adenomas and controls and between adenocarcinomas and controls. In adenomas, a distinct cytoplasmic presence of Dsg-2 was observed in addition to the usual membranous expression, with decreased expression in comparison with normal tissue. In malignant SGTs, Dsg-2 expression was absent. In most SGTs, beta4-integrin was expressed also with a distinct pattern, involving the cytoplasm and the unpolarised membrane, while CD44 was found only on the membrane. Strong correlation between beta4-integrin expression in adenomas and controls was noted, while CD44 expression was found to be correlated significantly between adenocarcinomas and controls (*p* < 0.001). Regarding ICAM-1, its expression was found increased in adenomas, with non-specific distribution in malignant SGTs and strong correlation between the histological subtypes and controls (*p* < 0.001).

**Conclusion:**

The different expression profile of CAMs in SGTs could possibly suggest a role on their pathogenesis, representing a model of how neoplastic cells can take advantage of normal tissue architecture and cell-extracellular matrix interactions.

## Background

The development and organization of multicellular organs, such as salivary glands, requires adhesion structures for intercellular communication, anchorage to the extracellular matrix (ECM), and triggering of intracellular pathways. This cell adhesion is mediated by subfamilies of transmembrane glycoprotein receptors named Cell Adhesion Molecules (CAMs) who mediate cell–cell and cell–matrix interaction and play important role in tissue morphogenesis, integrity and maintenance, as well as in immune responses [1–5]. Alteration of CAMs expression is associated with pathogenesis and progression of benign and malignant solid neoplasms of various tissues [6–10]. Desmoglein-2 (Dsg-2) is the main desmoglein expressed in desmosomal junctions (desmosomes) of mono/bilayer epithelia [11–13] (like salivary gland epithelium). Although the role of desmosomes in carcinogenesis is still unknown, previous studies have shown that Dsg-2 can act either as a tumor suppressor or an oncogene. Dsg-2 expression was upregulated in skin squamous cell carcinoma and head and neck cancer. In contrast, Dsg-2 expression was downregulated in pancreatic cancer, melanoma cells, and diffuse-type gastric cancer [14].

ICAM-1 is a cell-surface glycoprotein member of the immunoglobulin (Ig) superfamily. ICAM-1 is expressed on the surface of most cells and its expression is correlated with chronic inflammatory conditions like atherosclerosis, diabetes and cerebral malaria [15]. Previous studies have emphasised its role in elucidating cancer prognosis and progression by measuring the concentration of its soluble form as, for example, in advanced non-small cell lung cancer [16].

Moreover, beta4-integrin is also implicated in normal tissue epithelial-stromal architecture, neoplasm formation and immune responses by affecting cell cycle. Integrins are transmembrane receptors that facilitate cell-extracellular matrix adhesion; they form a critical connection between the extracellular matrix and the cell interior. Beta4-integrin electively binds with the alpha-6 subunit and then to laminin. In non-small cell lung cancer, tyrosine kinase receptor has been found to interact with integrin beta-4/alpha-6 ligand to facilitate tumor invasion, creating a microenvironment that promotes this type of cancer [17].

Pleomorphic adenomas (PAs) are the most common benign tumors of the salivary glands, which mainly arise from the major salivary glands, including the parotid and submandibular glands. Although most PAs are benign, recurrence may occur as well as malignant transformation. Previous studies have shown that CD44 cells can act as tumor inducing cells promoting tumor transformation [18].

So far, only a few studies have investigated through immunohistochemistry the expression and location of different CAMs in normal SGs and in specific types of benign or malignant tumours with contradictory results [4, 19–30]. The aim of this study was to determine a possibly altered expression profile (in comparison with normal tissue) of specific CAMs, including Dsg-2 (for the first time), beta4-integrin, CD44s and ICAM-1 in both benign and malignant SGTs, which could potentially affect intercellular and cell–matrix interactions, hence contributing to carcinogenesis.

## Methods

### Specimen

Tissue specimens were retrieved from 34 selected cases of adenomas, including pleomorphic adenoma (n = 30), myoepithelioma (n = 2) and oncocytoma (n = 2), and 31 cases of adenocarcinomas including acinic cell carcinoma (n = 7), epithelial-myoepithelial carcinoma (n = 3), adenoid cystic carcinoma (n = 3), polymorphous low-grade adenocarcinoma (n = 2), salivary ductal carcinoma (n = 3), mucoepidermoid (n = 3), squamous cell carcinoma (n = 5), adenocarcinoma not otherwise specified (NOS, n = 1), oncocytic carcinoma (n = 1), myoepithelial carcinoma (n = 1), and lymphoepithelial carcinoma (n = 2). Specimens were routinely fixed and embedded in paraffin and were retrieved from the archives of the Department of Histopathology of Red Cross Hospital in Athens, and Department of Oral Medicine/Pathology School of Dentistry, Aristotle University of Thessaloniki, Greece between 1999 and 2003. Diagnosis and classification were based on the criteria of the World Health Organization for salivary gland tumours [31]. In addition, 20 normal parotid and submandibular gland tissue adjacent to benign tumours and 7 normal minor labial salivary glands were also studied as controls.

### Immunohistochemistry and intensity of staining

The antigen retrieval and the Envision-HRP automated immunohistochemical technique (Dako Cytomation A/S, Glostrup, Denmark), using anti-CAMs antibodies (Santa Cruz, Biotechnology Inc, California, USA), were performed according to manufacturer instructions. Immunoreactivity was semi-quantitatively assessed and positivity was considered if greater than 5% of tumour cells were stained. Positivity was graded according to the percentage of tumour cells stained as I: 5–25%, II: 26–50%, III: 51–100%. The staining intensity was assessed as follows: negative (-), weak to moderate (+), moderate to strong (++) and strong (+++). Staining pattern for each neoplastic cell subpopulation was also characterized as cell membrane and/or cytoplasmic, whereas nuclear staining was not considered as positive. Sections were examined by three of the authors (CB, AE, DA) independently. Sections were re-examined when there were differences, and discussion was occasionally necessary to establish uniformity. Patients had given informed consent, and the whole study was performed according to the declaration of Helsinki II.

### Statistics

Rank correlation tests were performed for the type of tissue (taking in pairs: control–adenoma, control–adenocarcinoma, adenoma–adenocarcinoma) and the observed intensity of staining. Furthermore, a similar analysis was performed for the type of tissue and the observed positivity. Non-parametric tests, Kendall’s τ and Spearman’s ρ were used for statistical analysis. Results were processed using SPSS ver. 25 (IBM, USA).

## Results

### CAMs in normal salivary glands and benign SGTs

**Fig. 1:**
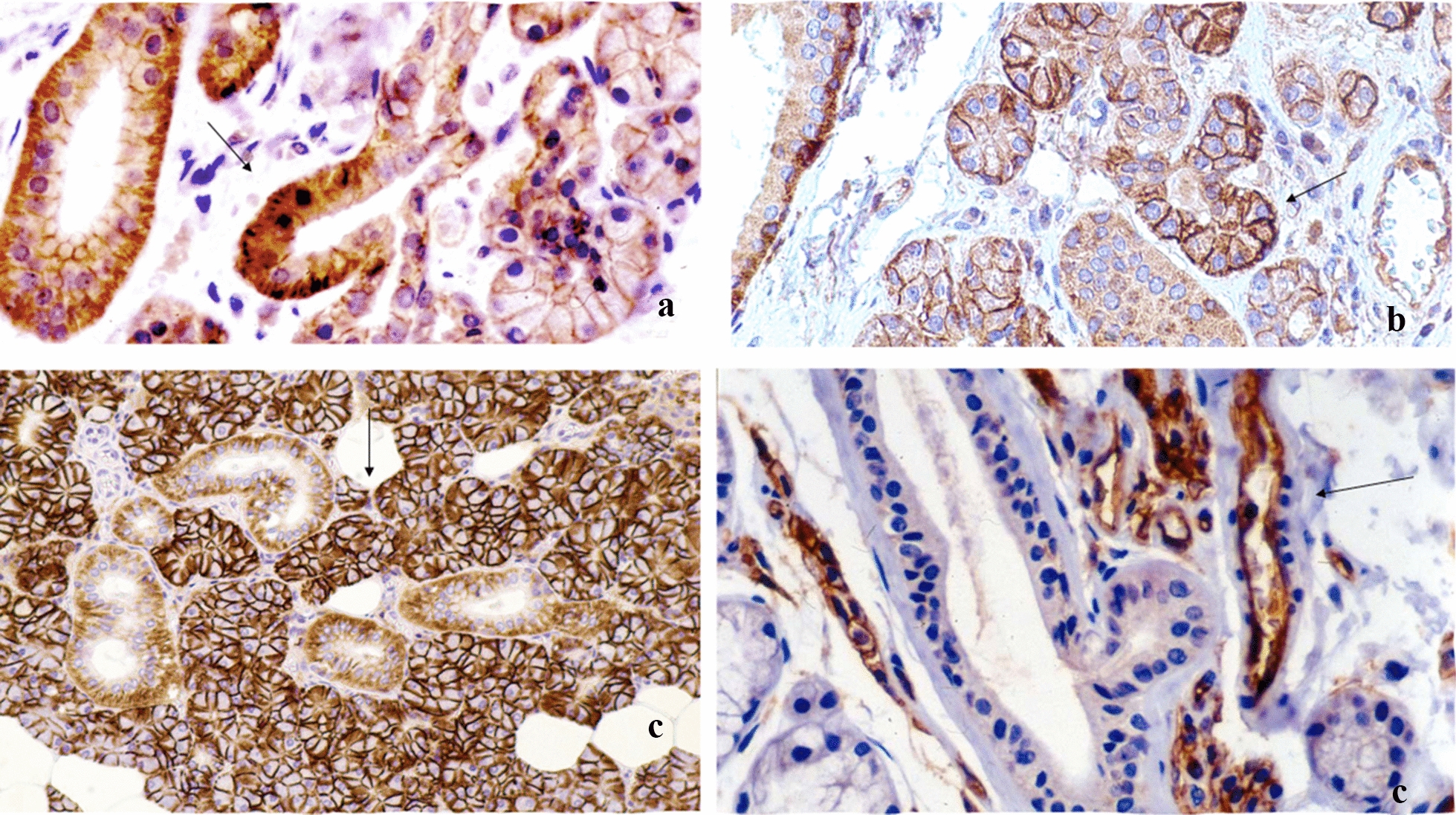
Cell adhesion molecules in normal salivary gland tissue. **a** Desmoglein-2 (Dsg-2): Linear membranous staining of acinar and ductal cells. **b** Beta4-integrin: Strong immunoreactivity of basolateral portion of serous cell membrane and basal cells of interlobular ducts. **c** HCAM (CD44s): Strong membranous staining of acinar cells (mainly serous), decreased expression in basolateral surfaces of ductal basal cells and partially in stromal non-epithelial cell subpopulations. **d** ICAM-1: Negative staining of epithelial cells. Membranous staining of non-epithelial stromal subpopulations such as endothelium, lymphocytes and fibroblasts

Immunohistochemical results for all examined CAMs were summarized in Table 1 and for benign SGTs in Table 2. In all cases, Dsg-2 was expressed at epithelial cell to cell contacts of acinar and ductal cells but was absent from their luminal-apical surface as well as from basal surface that participates in cell-basement membrane connections. Τhe staining of this receptor was more intense mainly in the ducts than the acini and on basal cells rather than their apical pole (Fig. 1a) while Dsg-2 was not expressed in stromal cells. Regarding beta4-integrin, it was intensely (+++) expressed mainly on the basolateral surface of acinar (serous and mucous), myoepithelial cells, intercalated ductal cells and basal cells of excretory ducts (Fig. 1b). Endothelial cells were also positive for beta4-integrin expression, whereas the rest stromal cell subpopulations were negative. CD44s receptor was strongly expressed on the surface of acinar cells, mainly serous, in all cases. Staining in ductal epithelium was moderate, involving the basolateral surfaces of basal cells, and focally the intercellular contacts of luminal cells (Fig. 1c). Stromal lymphocytes were also positively stained for CD44s. ICAM-1 was absent from the normal glandular epithelium but was expressed in the endothelial and stromal cells including a few inflammatory cells (lymphocytes and macrophages) (Fig. 1d).

**Table 1:** Qualitative and quantitative pattern of cell adhesion molecules expression in normal salivary gland tissues

Localization–Immunoreactivity, Intensity of staining										
CAMs	Epithelial cells	Acini		Intercalated ducts	Striated duct		Excretory ducts		Endothelium	Stromal cells
		Luminal	Non luminal	Luminal	Luminal	Non luminal	Luminal	Non luminal		
Dsg–2	III (27/27)	++	++	+++	++	+++	++	+++	–	–
Beta4–Integrin	III (27/27)	++	+++	+++	+++	+++	+	+++	++ (27/27)	–
CD44s	III (16/27) II (11/27)	+++	+++	+++	+	++	+	++	++ (27/27)	++ II (27/27)
ICAM-1	–	–	–	–	–	–	–	–	+++ (27/27)	+++ II (27/27)

**Table 2:** Expression of cell adhesion molecules in benign SGTs

CAMs	Positivity (%)	Pattern of staining	Topography and intensity of staining			
			Luminal cells	Non-luminal cells	Cells in Islands	Solitary neoplastic stromal cells
Dsg-2	III (30/30)	C (mainly)/M	++	+	++	–
Beta4-integrin	III (30/30)	M (mainly)/C	++	++	++	++
CD44s	III (30/30)	M	+	++	++	–
ICAM-1	III (8/30) II (13/30) I (9/30)	M: (Luminal surface mainly)/C	+	–	+	+
Myoepitheliomas						
CAM	Positivity	Pattern of staining	Intensity of staining			
Dsg-2	III (2/2)	C	++			
Beta4-integrin	III (2/2)	M/C	+++			
HCAM (CD44s)	II: 2/2	M	++			
ICAM-1	III: 1/2 I: 1/2	C	+ ++			
Oncocytomas						
CAM	Positivity	Pattern of staining	Intensity of staining			
Dsg-2	III: 2/2	C	++			
Beta4-integrin	III: 2/2	M/C	+++			
HCAM (CD44s)	III: 2/2	M	+			
ICAM-1	–	C	+

### Pleomorphic adenoma

**Fig. 2:**
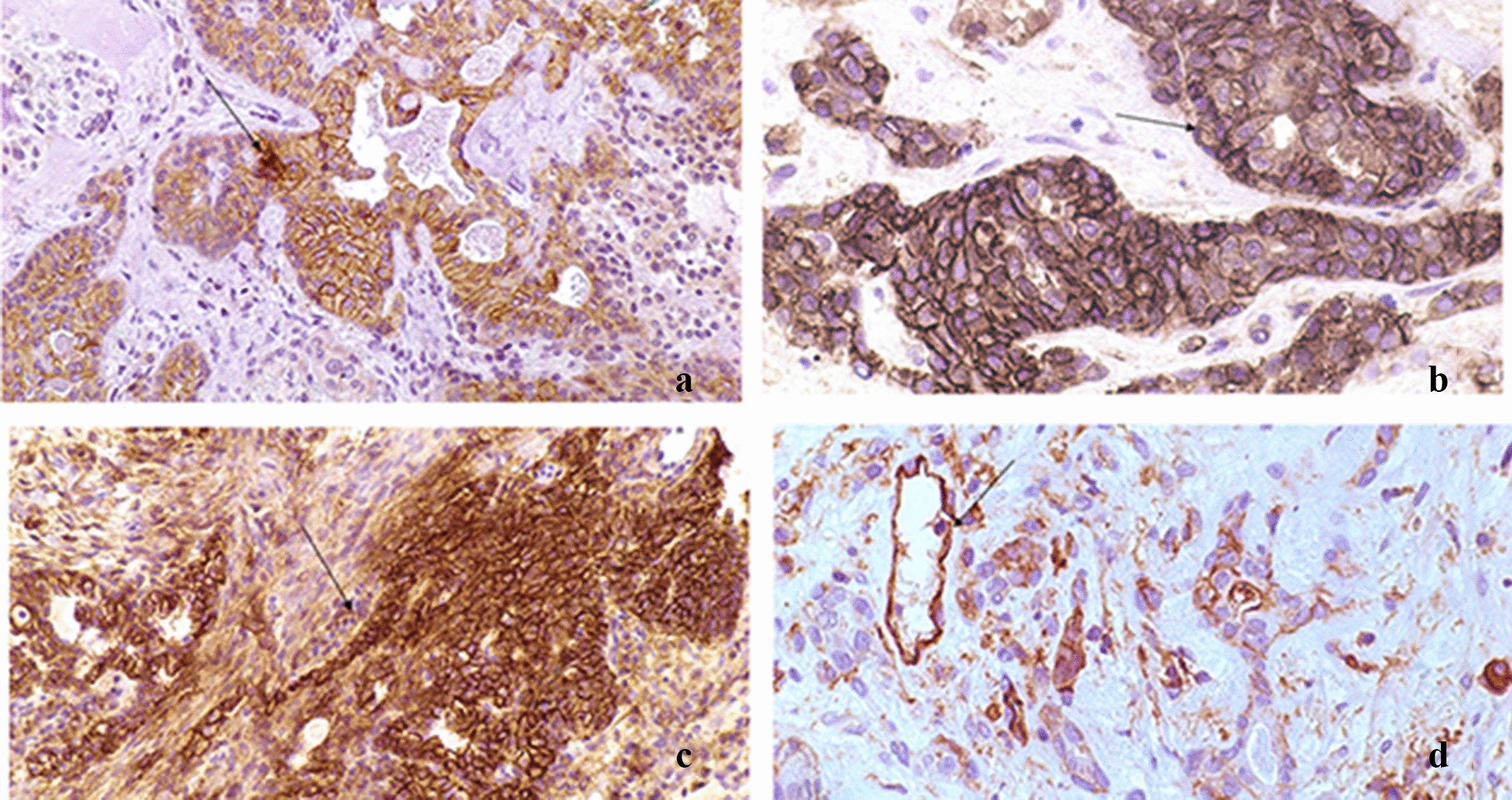
Cell adhesion molecules in Pleomorphic Adenoma. **a** Dsg-2: Membranous or diffuse cytoplasmic staining at the intercellular contacts of neoplastic cells of all architecture types except luminal surfaces of luminal cells and basal surfaces of non-luminal cells. No positivity in stromal cells (Envision/HRP × 100). **b** Beta4-integrin: Strong membranous positivity at cell-basal membrane contacts, intercellular connections of the great majority of cells and stromal cells (Envision/HRP x200). **c** HCAM (CD44s): Strong membranous expression in the majority of neoplastic cells of all structures especially in stromal cells, cells of solid islands, non-luminal cells and intercellular contacts between luminal and non-luminal cells (Envision/HRP x200). **d** ICAM-1: Fibroblastoid stromal cells mainly in myxoid areas, linear positivity at the luminal surface of luminal ductal cells and moderate expression in some cells of solid islands (Envision/HRP x200). Strong immunoreactivity of basolateral portion of serous cell membrane and basal cells of interlobular ducts

Dsg-2 was found in all cases showing mainly a cytoplasmic pattern in more than 50% of neoplastic cells, excluding solitary plasmacytoid cells in myxoid stroma (Fig. 2a). Beta4-integrin showed a diffuse, strong positivity at both cell-membrane and cytoplasm in more than 50% of neoplastic cells in all neoplastic structures (Fig. 2b). In all cases, CD44 was expressed along the membrane of intercellular and cell–matrix contacts of most neoplastic cells, especially in cells of solid structures, cells showing stromal differentiation and non-luminal cells of duct-like structures (Fig. 2c). Lymphocytes, endothelial, and neoplastic cells, located mainly in myxoid stroma were positive for ICAM-1. Moreover, in some of the cases, a continuous positive linear staining on the luminal surface of luminal cells in neoplastic duct-like structures was observed (Fig. 2d).

### Myoepithelioma and oncocytoma

In both myoepithelioma and oncocytoma most of neoplastic cells showed a moderate cytoplasmic staining for Dsg-2 whereas beta4-integrin was strongly expressed among the cell membrane and cytoplasm of neoplastic cells. However, the number of positive cells for CD44s in myoepithelioma was less than in oncocytoma (10–25% and > 50%). However, the intensity of staining for oncocytoma was weak. Finally, ICAM-1 was absent in oncocytoma, whereas the two cases of myoepitheliomas showed a heterogeneous positivity.

### CAMs in malignant SGTs (adenocarcinomas)

The presence of beta4-integrin, Dsg-2, ICAM-1 and HCAM (CD44s) in malignant tumors was presented in Table 3, emphasizing the localization and intensity of staining in each tumor type. In all cases, beta4-integrin had a moderate to strong expression in unpolarized cell membrane, different to its normal distribution on the basolateral surfaces of cells.

**Table 3:** Expression of cell adhesion molecules ICAM-1 and HCAM (CD44s) in malignant carcinomas

Type of tumor	ICAM-1			HCAM (CD44s)		
	Positivity	Pattern	^Intensity^	Positivity	Pattern	Intensity
ACC (n = 7)	III (1) I (6)	M M (3), M (luminal surface) (3)	+++ ++	III	M	+++
EMYC (n = 3)	II (2) I (1)	M (luminal cells/surface) M (non-luminal cells)	++	I	M (non-luminal cells)	+++
ADCC (n = 3)	(-) (2), I (1)	M	++	III	M	++
PLGA (n = 2)	II	M (luminal surface of luminal cells)	++	II	M (luminal central cells)	+++
MEC (n = 3)	II	M	++	III	M(epithelioid/intermediate cells)	+++
SDC (n = 3)	III (2) I % (1)	C M/C	++ +++	III (1) I (2)	M	+++ ++
SCC (n = 5) High grade (2) Low grade (3)	I III (1), I (2)	M	++	I III	M	+ +++
LEPC (n = 2)	I (1), II (2)	M	+++	I (1), II (2)	M	++ , +++
ONC (n = 1)	0	M	++	II	M	++
MYEC (n = 1)	I	M	++	III	M	++
ANOS (n = 1)	III	M	++	0	M/C	+

*ΑCC* Acinic cell carcinoma, *EMYC* Epithelial-myoepithelial carcinoma, *ADCC* Adenoid cystic carcinoma, *PLGA* Polymorphous low-grade adenocarcinoma, *MEC* Mucoepidermoid carcinoma, *SDC* Salivary ductal carcinoma, *SCC* Squamous cell carcinoma, *LEPC* Lymphoepithelial carcinoma, *ONC* Oncocytic carcinoma, *MYEC* Myoepithelial carcinoma, *ANOS* Adenocarcinoma not otherwise specified, *n* Number of cases, *M* Membranous, *C* Cytoplasmic

Regarding Dsg-2, the intensity of staining was strong (+++) in 100% of control samples, while in adenomas, 100% of specimens were stained moderately (++). In malignant cases, 77% of cases (24/31) had moderate intensity of staining, while in 10% (3/31) there was weak staining. In 13% of all malignancies, staining was negative (-). Very strong correlation was found in Dsg-2 staining expression between controls and adenomas (Kendall’s τ-c = 0.987, *p* < 0.001; Spearman’s ρ = 1, *p* < 0.001; Table 4) and between controls and malignancies (Kendall’s τ-c = 0.995, *p* < 0.001; Spearman’s ρ = 0.950, *p* < 0.001; Table 4). Furthermore, in malignant tumors, results of Dsg-2 revealed severe decrease or loss of membrane expression, which was related to the type of malignancy. A cytoplasmic and even membranous expression was noticed in most neoplastic cells in acinic cell carcinoma, epithelial-myoepithelial carcinoma, adenoid cystic carcinoma, polymorphous low-grade adenocarcinoma, salivary ductal carcinoma, squamous cell carcinoma, whereas was focally presented or absent in adenocarcinoma NOS, oncocytic carcinoma, myoepithelial, and lymphoepithelial carcinomas.

**Table 4:** Correlation between different kind of cells and Intensity of staining

CAM	Type off cells	N	Kendall’s τ-c	Spearman’s ρ	Kendall’s τ-c significance	Spearman’s ρ significance
Dsg-2	Control–Adenoma	61	0.987	1.000	< 0.001	< 0.001
	Control–Adenocarcinoma	58	0.995	0.950	< 0.001	< 0.001
Beta4-Integrin	Control–Adenoma	61	0.871	0.877	< 0.001	< 0.001
	Control–Adenocarcinoma	58	0.193	0.317	0.006	0.015
CD44s	Control–Adenoma	61	0.058	0.164	0.145	0.206
	Control–Adenocarcinoma	58	0.578	0.579	< 0.001	< 0.001
ICAM-1	Control–Adenoma	61	0.987	0.990	< 0.001	< 0.001
	Control–Adenocarcinoma	58	0.995	0.967	< 0.001	< 0.001

Regarding beta4-integrin, intensity of staining was highly correlated between adenomas and controls (Kendall’s τ-c = 0.871, *p* < 0.001; Spearman’s ρ = 0.877, *p *< 0.001; Table 4), while malignancies were similar with controls (Table 4). More specifically, while strong (+++) intensity of staining had been observed in 100% of controls, only 12% (4/34) of benign adenomas had had the same feature. In malignancies, on the other hand, 81% (30/34) were strongly (+++) stained.

Regarding CD44, intensity of staining was not significantly correlated between controls and benign adenomas (Table 4). However, controls and adenocarcinomas were significantly correlated (Kendall’s τ-c = 0.578, *p* < 0.001; Spearman’s ρ = 0.579, *p *< 0.001; Table 4). All controls and 94% (32/34) of benign adenomas have been moderately stained (++) for CD44, while in malignant cases 21 out of 31 cases (68%) have been strongly (+++) stained for the same antibody. In seven out of 31 cases (23%) moderate (++) staining was noticed, while three out of 31 (10%) had presented weak intensity.

Regarding ICAM-1, strong correlation was noted between controls, adenomas and adenocarcinomas (*p* < 0.001).

## Discussion

Desmosomes, hemidesmosomes and other intercellular and cell–matrix connections are playing an important role in tissue homeostasis and tissue architecture through cell to cell interaction [1]. To the best of our knowledge, the expression of desmosomal component Dsg-2 in salivary gland tissues was only generally referred in a study of Schäfer et al. [11]. In our study we identified the exact pattern of Dsg-2 expression in glandular epithelium as a membrane intercellular connector of all acinar and ductal cells, mainly at the basal pole of acinar and myoepithelial cells and apical pole of luminal cells of excretory ducts.

Limited and conflicting information of different CAMs in the development of pleomorphic adenoma were reported in the literature [27, 28, 32–42]. Furthermore, to our knowledge, Dsg-2, the main desmosomal cadherin in salivary glandular epithelium has not been investigated in depth so far [43]. According to our results, Dsg-2, showed decreased and alternative, mainly cytoplasmic, expression in most of the neoplastic cells excluding the solitary plasmacytoid cells in myxoid stroma. These findings were similar to the abnormal, decreased expression that was reported in gastric and breast adenocarcinomas [44, 45], and suggest that the loss of intercellular desmosome contacts could possibly be an important event during salivary gland neoplastic development. In salivary gland pleomorphic adenoma, this cell to cell detachment may be partially associated with the increased stromal component secretions, which extend the intercellular space breaking the intercellular junctions.

Few papers have evaluated the presence of CAMs subspecies in normal salivary glands. Loducca et al. [35] have described the normal topography of hemidesmosomal beta4-integrin in normal salivary gland tissues. Our study was in agreement with previous data, suggesting that beta4-integrin was strongly expressed at the basolateral membranous portions of serous and mucous acinic cells indicating the existence of laminin not only in basement membrane, but also in intercellular space function as a cell–cell bridge. Loducca et al. [35] also found that beta4-integrin distributed in well-developed clusters on luminal cell pole of intercalated ducts, in a delicate bipolar manner in the striated ducts, and limited to a few intercellular contacts and cell-membrane junctions of excretory ducts. However, we observed strong expression of beta4-integrin at the basolateral surface of intercalated ducts and the overall basal cell membrane of striated and excretory ducts. This can possibly suggest that beta4-integrin and laminin are involved in cell-basement membrane and possibly intercellular connections of ductal epithelium.

There are previous studies, like that of Franchi et al. [29, 41], that had investigated the expression of integrin in pleomorphic adenoma (especially integrin a-subunits) and its participation in cell–cell contacts of tubular and ductal structures, cell–matrix connections and isolated cells in stroma. Furthermore, Lourenço et al. [28] found that integrin a-subunits were intensely expressed on neoplastic luminal cells. Beta-1 subunit was also found to be widely expressed on luminal cells of neoplastic ducts and neoplastic modified myoepithelial cells were positive for a subunit and beta-1 subunit expression at their intercellular contacts. Sunardi-Widyaputra and Van Damme [46] have investigated the expression of beta-1 and beta4-integrins in pleomorphic adenomas. Beta 4-integrin was found again strongly positive on the luminal cells of duct-like structures and their basement membrane. In our study, we found strong immunoreactivity for beta4-integrin in cells of all neoplastic structures in accordance with the above studies. This finding may be associated with the abundant cellular secretion of laminin (the main beta4-integrin ligand) and depositions in stroma [22, 47–49]. Normally, beta4-integrin participates in junctions between cells and laminin of basement membrane via α6β4 heterodimer in hemidesmosomes. In pleomorphic adenomas the pattern of beta4-integrin expression is supporting its role in intercellular contacts and could also be associated with cell movement and migration by binding the stromal laminin, a theory that had been originally proposed by Mercurio and Rabinovitz [50].

Instead of the limited expression of HCAM (CD44s) in myoepithelial and rarely in ductal cells that observed by Xing et al. [26], many other studies have detected a more intense expression for HCAM (CD44s) and its isoforms in salivary gland tissues [27, 36, 37, 51]. Similarly, we found strong membranous immunoreactivity in acinar cells (mainly serous), which decreased in intercalated and striated ductal cells. This pattern of expression may be associated with the different hyaluronate or other matrix molecules expression, which act as ligands for HCAM (CD44s) in salivary gland tissues. Interestingly, lymphocytes and macrophages showed positive membranous staining due to the existence of abundant hyaluronate in stroma [26]. These findings suggested a dual role for CD44s, mediating in intercellular and cell–matrix interactions. In pleomorphic adenoma, the expression of CD44s was investigated by Franchi et al. [37] and Xing et al. [26]. Similarly to their findings, we observed a strong immunoreactivity of this receptor in neoplastic cells of every structure (tubular, ductal, solid, isolated cells in myxoid stroma), associated with the excessive presence of hyaluronate, osteopontin and other stromal components, which are the main HCAM (CD44s) ligands [2, 52].

In a study of Perschbacher et al. [27] a diffuse cytoplasmic pattern of staining for ICAM-1, in ductal and sporadically in serous acinar cells had been observed. On the other hand, our results, similarly to Kapsogeorgou et al. [39] and Flipo et al. [40] indicated that in normal salivary gland tissues, ICAM-1 is almost absent from both acinar and ductal epithelium, being present in endothelium, fibroblasts around the ducts and a small number of lymphocytes and monocytes. ICAM-1 had only been examined in few cases of pleomorphic adenoma with insufficient results [27]. According to our results, ICAM-1 showed a linear pattern of immunoreactivity at the luminal-apical pole of luminal cells in neoplastic duct-like structures resembling salivary ducts in sialadenitis. This finding together with ICAM-1 immunoreactivity for endothelium and lymphocytes, may indicate an activation of immunologic mechanisms by neoplastic epithelium to restrict neoplastic transformation and tumour formation. On the other hand, the presence of ICAM-1 in some neoplastic cells located in the stroma, may indicate its participation in tumour cell spreading. The pattern of ICAM-1 expression is also associated with the stromal depositions, as ICAM-1 positive stromal cells were mostly observed in myxoid rather than chondromyxoid stroma.

According to our results, the pattern of the expression of the above described CAMs represents their participation in normal salivary gland architecture and supports a distinct profile of acinar and ductal epithelial subpopulations based on their luminal or non-luminal stratification. The pattern of the examined cell adhesion molecules in pleomorphic adenomas, consisted of qualitative and quantitative alterations of Dsg-2, the unpolarised staining of laminin-receptor beta4-integrin, and the abundant presence of hyaluronate receptor HCAM (CD44s), supporting the idea that this neoplasm resembles a defect form of salivary gland embryogenesis. At this point we would like to emphasize the limitations of our study which are the limited number of other subtypes of benign salivary gland tumors as most of our specimen were pleomorphic adenoma and the use of immunohistochemistry only rather than RT-PCR. It may be hypothesised that unknown genetic or epigenetic factors may evaluate an uncontrolled overproduction of stromal components and abnormal, failed, and incomplete formation of cellular structures that consisted of modified but not entirely differentiated ductal and myoepithelial neoplastic cells, bearing CAMs with altered function. In this phase, intercellular connections are disrupted (decrease or loss of Dsg-2), other receptors help in cell motivation and migration (beta4-integrin, HCAM-CD44s and perhaps ICAM-1), connective tissue is produced and organised, and cell-stroma interactions are formed (HCAM-CD44s) in order to assist tissue growing and differentiation. Finally, the activation of immune system (with participation of ICAM-1) is trying to control and to restrict the neoplastic transformation and cell spreading, in contrast to salivary gland malignancies, in which ICAM seems to help neoplastic cell migration and invasion rather than control them [53].
